# Divergent molecular events underlying initial T-cell commitment in human prenatal and postnatal thymus

**DOI:** 10.3389/fimmu.2023.1240859

**Published:** 2023-09-27

**Authors:** Han He, Yingpeng Yao, Lindong Tang, Yuhui Li, Zongcheng Li, Bing Liu, Yu Lan

**Affiliations:** ^1^ Key Laboratory for Regenerative Medicine of Ministry of Education, Institute of Hematology, School of Medicine, Jinan University, Guangzhou, China; ^2^ Basic Medicine Postdoctoral Research Station, Jinan University, Guangzhou, China; ^3^ State Key Laboratory of Experimental Hematology, Haihe Laboratory of Cell Ecosystem, Institute of Hematology, Senior Department of Hematology, Fifth Medical Center of Chinese PLA General Hospital, Beijing, China

**Keywords:** human thymus, scRNA-seq, T cell development, double-negative thymocytes, alternative polyadenylation

## Abstract

**Introduction:**

Intrathymic T-cell development is a coordinated process accompanied by dynamic changes in gene expression. Although the transcriptome characteristics of developing T cells in both human fetal and postnatal thymus at single-cell resolution have been revealed recently, the differences between human prenatal and postnatal thymocytes regarding the ontogeny and early events of T-cell development still remain obscure. Moreover, the transcriptional heterogeneity and posttranscriptional gene expression regulation such as alternative polyadenylation at different stages are also unknown.

**Method:**

In this study, we performed integrative single-cell analyses of thymocytes at distinct developmental stages.

**Results:**

The subsets of prenatal CD4^–^CD8^–^ double-negative (DN) cells, the most immature thymocytes responsible for T-cell lineage commitment, were characterized. By comprehensively comparing prenatal and postnatal DN cells, we revealed significant differences in some key gene expressions. Specifically, prenatal DN subpopulations exhibited distinct biological processes and markedly activated several metabolic programs that may be coordinated to meet the required bioenergetic demands. Although showing similar gene expression patterns along the developmental path, prenatal and postnatal thymocytes were remarkably varied regarding the expression dynamics of some pivotal genes for cell cycle, metabolism, signaling pathway, thymus homing, and T-cell commitment. Finally, we quantified the transcriptome-wide changes in alternative polyadenylation across T-cell development and found diverse preferences of polyadenylation site usage in divergent populations along the T-cell commitment trajectory.

**Discussion:**

In summary, our results revealed transcriptional heterogeneity and a dynamic landscape of alternative polyadenylation during T-cell development in both human prenatal and postnatal thymus, providing a comprehensive resource for understanding T lymphopoiesis in human thymus.

## Introduction

While commitment to most blood cell lineages occurs primarily in the bone marrow and/or spleen, T lineage commitment depends on the specific microenvironment of the thymus. In humans, T-cell development is initiated by settling of thymus-seeding progenitors (TSPs) from fetal liver as early as gestational week 7 (1). From gestational week 22 onward, TSPs from the bone marrow become the source of thymic immigrants (2). These TSPs then develop into early thymic progenitors (ETPs) and undergo a strict T-cell commitment process. Uncommitted ETPs have multilineage (e.g., myeloid cell, dendritic cell, natural killer cell, B lymphocyte, and erythrocyte, in addition to T lymphocyte) potential, and commitment to the T-cell fate is in concert with loss of potential for other lineages (3, 4). From here onward, committed thymocytes begin to rearrange their T-cell receptor (TCR) mediated by RAG proteins and finally differentiate into either αβ T cells or γδ T cells (5). Similar to mice, human αβ T thymocyte development processes through a series of coordinated and successive stages that can be characterized based on specific surface receptor expression, starting from CD4^–^CD8^–^ double-negative (DN) thymocytes, followed by CD4^+^CD8^–^ immature single-positive (ISP) and CD4^+^CD8^+^ double-positive (DP) populations, culminating with CD4^+^CD8^–^ or CD4^–^CD8^+^ single-positive (SP) cells. The most immature thymocytes are DN cells and can be subdivided into three populations (DN1–DN3) characterized by the expression of CD34, CD38, and CD1a in the postnatal thymus (6). DN1 cells express CD34 but lack CD1a and CD38 expression. Transition from DN1 to DN2 stage is accompanied by the upregulation of CD38, following by differentiation of DN3 cells expressing CD34 and CD38 as well as CD1a.

Although considerable advances have been made toward understanding human T-cell development, several major questions remain obscure regarding the heterogeneity and molecular mechanisms underlying the fate bifurcation of certain T progenitors. Several laboratories have largely resolved these long-standing questions by using single-cell RNA sequencing (scRNA-seq) technologies to study human T-cell development in both the developing fetus and postnatal thymus (7–13). Our group first revealed thymus organogenesis, early T lymphopoiesis, and their pre-thymic lymphoid progenitors in the early human fetus at single-cell resolution (7, 14). Following studies further transcriptomically characterized the TSPs and revealed T lineage specification, commitment, and subsequent differentiation in both the prenatal and postnatal thymus (8, 10, 11). A recent work profiled the complete thymocyte populations in postnatal samples, especially the three CD4^–^CD8^–^ DN subsets and identified three distinct TSP subpopulations as well as revealed T-cell lineage commitment and divergence of non-T-cell lineages (12). Despite that the characteristics of immature DN subsets have been characterized in the human postnatal thymus (11, 12), less is known regarding the nature of distinct DN subpopulations and cell fate decision (T lineage versus non-T lineage) during DN stages of T-cell development in human fetus. Furthermore, mouse studies have well documented that fetal and adult DN cells (including ETP) are not equivalent, reflecting the origin of ETP (HSC-dependent or HSC-independent), thymic outputs, differentiation speed, and gene expression transition (15, 16), so whether these differences existing between prenatal and postnatal human thymocytes are also worth to be addressed.

Alternative polyadenylation (APA) is an RNA-processing mechanism that generates a diversity of mRNA isoforms through differential usage of distinct polyadenylation sites (17). APA has been observed in over 70% of mammalian mRNA-encoding genes and occurs most frequently in the 3’ untranslated region (3’ UTR) of mRNAs (18–20). Alternative 3’ UTR isoforms interact with RNA-binding proteins and/or microRNA to modulate posttranscriptional regulatory mechanism by affecting mRNA stability, translation, nuclear export and localization, and protein localization (17, 21). Here, 3’ end sequencing or bulk RNA-seq has revealed the global landscape of APA in all eukaryotes, and APA is dynamically regulated in a tissue- and/or cell type-specific manner (22–25). With the advance of scRNA-seq technology, the single-cell landscape of APA events in various tissues, biological processes, and diseases has been extensively exploited (26–29), contributing to the understanding of dynamic gene expression regulation at greater cell type and isoform resolution. However, to date, the cell-to-cell heterogeneity in APA usage and APA dynamics across human T-cell development is still unexplored.

To explore the cellular heterogeneity in the developing and functionally mature thymus, we constructed an integrative transcriptomic atlas of human thymocytes involving both prenatal and postnatal stages. Specifically, we disclosed the following aspects: 1) characterizing the prenatal DN subpopulations transcriptomically; 2) revealing the transcriptional differences between prenatal and postnatal DN cells regarding key gene expressions, enriched GO terms, and metabolic programs; and 3) illustrating the different preferences of polyadenylation site usage in prenatal and postnatal thymocytes at single-cell resolution.

## Results

### Integrated single-cell transcriptomics of human thymocytes

**Figure 1:**
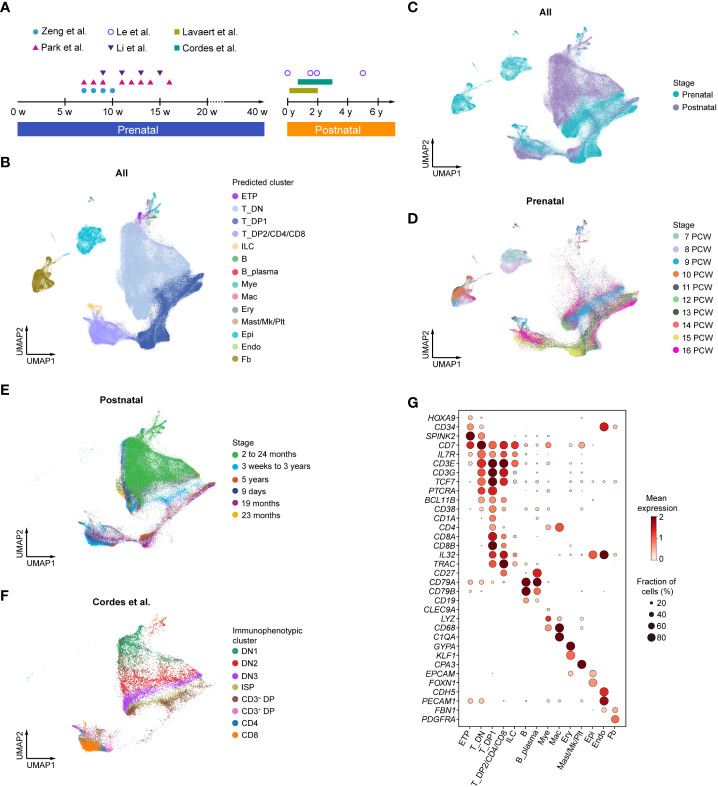
Integrative analysis of human thymocytes. **(A)** Summary of gestational stage/age of samples from six published datasets. Each symbol (circle or triangle) denotes an individual time point, while the bar represents the corresponding time frame. **(B)** Visualization of uniform manifold approximation and projection (UMAP) for cellular composition of human thymus colored by cell type (ETP, early thymic progenitor; DN, double-negative T cell; DP, double-positive T cell; ILC, innate lymphoid cell; Mye, myeloid cell; Mac, macrophage; Ery, erythrocyte; Mk, megakaryocyte; Plt, platelet; Epi, epithelial cell; Endo, endothelial cell; Fb, fibroblast). **(C)** Visualization of UMAP for cellular composition of human thymus colored by prenatal and postnatal group. **(D)** Visualization of UMAP for cellular composition of human prenatal thymus colored by postconception weeks (PCW). **(E)** Visualization of UMAP for cellular composition of human postnatal thymus colored by different age groups. **(F)** Visualization of UMAP for cellular composition of human postnatal thymus colored by immunophenotypic subsets. The dataset was derived from Cordes et al. (12). **(G)** Dot plot showing feature gene expression in each cell cluster.

To comprehensively understand the transcriptomic landscape of whole development stages/events of human thymic T cells in both prenatal and postnatal thymus, we performed integrative analysis of several published scRNA-seq datasets (7–12) of the human thymus, ranging from 7 postconception weeks (PCW) to 16 PCW (prenatal) and 9 days to 5 years (postnatal) (**Figure 1A**, **Supplementary Table S1**). A total of 187,842 cells from developing and postnatal thymus were pooled together for downstream analysis. A two-dimensional uniform manifold approximation and projection (UMAP) was then used to visualize the global transcriptional changes in the thymus. Using CellTypist (30) with built-in models, 14 cell clusters were readily annotated according to the expression of specific marker genes. Differentiating T cells were well represented in both prenatal and postnatal datasets, including ETP, DN, DP, and CD4/CD8 T cells (**Figures 1B–G**).

### Characterization of human prenatal DN subpopulations *in silico*

**Figure 2:**
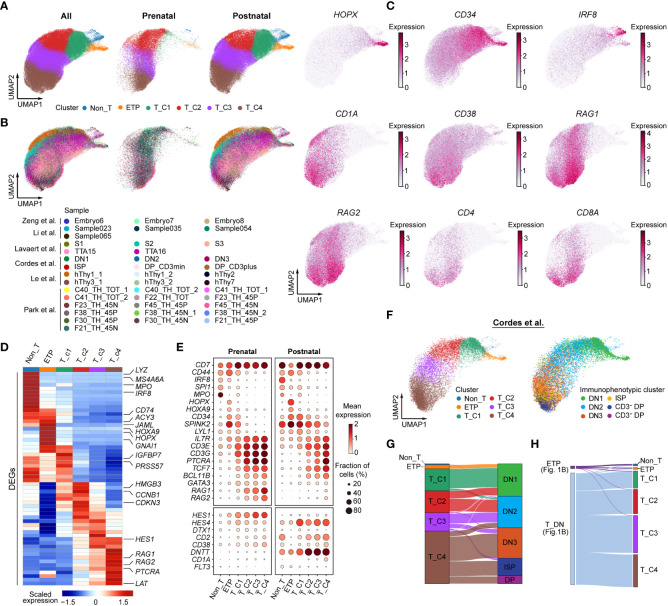
Identifying human prenatal double-negative (DN) subpopulations transcriptionally. **(A)** UMAP visualization of distinct subclusters of DN cells. **(B)** UMAP plot showing DN subclusters colored by different sources. **(C)** UMAP plot showing *HOPX*, *CD34*, *IRF8*, *CD1A*, *CD38*, *RAG1*, *RAG2*, *CD4*, and *CD8A* gene expression. **(D)** Heatmap showing blocks of differentially expressed genes (DEGs; top 10) by six subclusters of human thymic DN cells. **(E)** Dot plots depicting the expression of selected genes in prenatal (left) and postnatal (right) DN subclusters. **(F)** UMAP representing DN subclusters from Cordes et al. (12). **(G)** Sankey plots showing the proportion of DN subclusters with immunophenotypic characteristics. **(H)** Mapping the identified six subclusters with the populations depicted in **Figure 1B**.

A recent study has reported the transcriptional characteristics of distinct DN subpopulations in human postnatal thymus (12), whereas the cellular heterogeneity of prenatal DN cells has not been well deciphered. To identify DN subpopulations within the context of the developing thymus, we next fetched all integrated DN cells for further analysis. A total of 24,467 prenatal cells and 71,720 postnatal cells were included. After cell cycle regression and dimensionality reduction, these cells were further divided into six subclusters and annotated as Non_T, ETP, T_C1, T_C2, T_C3, and T_C4 (**Figures 2A, B**). The Non_T cluster was identified by the high expression of plasmacytoid DC marker *IRF8* and myeloid marker *MPO*. ETP was characterized based on the expression of hematopoietic progenitor genes *CD34*, *HOPX*, and *HOXA9* and alternative lineage genes (*IRF8*, *SPI1)*. Of note, *HOPX* and *HOXA9* were specifically highly expressed only in ETP but not adjacent Non_T or T_C1 clusters. The T_C1 cluster still retained stem/progenitor features (*CD34*, *SPINK2*) and alternative lineage potential (*MPO*, *SPI1*). The T_C2 cluster showed declined expression of genes for stem/progenitor cells and alternative lineage cells and started to express some T lineage genes at a low level. The T_C3 cluster further downregulated the expression of stem/progenitor genes and upregulated the expression of TCR rearrangement genes (*RAG1*, *RAG2*, and *PTCRA*). The T_C4 cluster showed the highest expression of TCR rearrangement genes, T-cell lineage genes (*TCF7*, *BCL11B*), and *CD4* and *CD8A*, suggesting that T-cell lineage specification and commitment progressed (**Figures 2C–E**). The pattern of sequential expression of signature genes in prenatal and postnatal clusters was almost identical (**Figure 2E**). Nevertheless, the expression of some key T-cell genes was not consistent between prenatal and postnatal clusters. For example, the expression of *RAG1* and *RAG2* was as early as in prenatal T_C1 cluster but only in postnatal T_C4 cluster (**Figure 2E**). Cordes et al. (12) previously used flow cytometry to sort all postnatal thymocyte populations based on surface marker expression and subjected them to scRNA-seq analysis. We next explored the corresponding relationship of these identified clusters with their immunophenotypic clusters. Non_T, ETP, T_C1, and a small part of the T_C2 cluster belonged to DN1 cells. The other part of T_C2 and the majority of the T_C3 cluster fell into DN2 cells. The T_C4 cluster was a mixture population, including DN3, ISP, and DP cells (**Figures 2F, G**). We also mapped the identified six clusters with the populations depicted in **Figure 1B**. The Non_T and ETP subclusters were almost mapped to the ETP population; the remaining subclusters corresponded to DN cells (**Figure 2H**).

**Figure 3:**
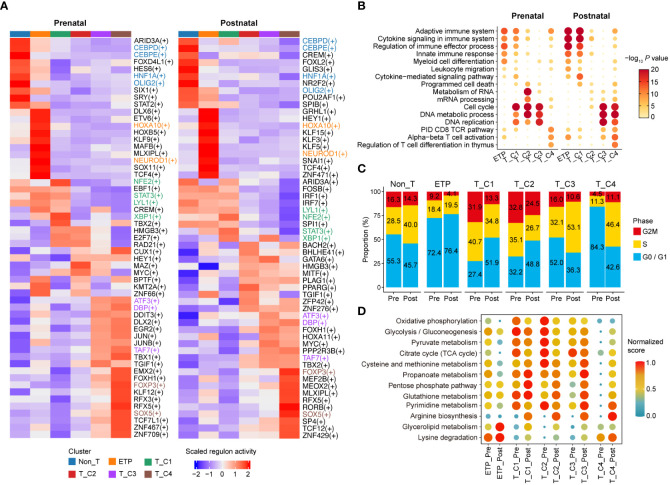
Delineating transcriptional characteristics of DN subclusters. **(A)** Heatmap showing the enriched regulons in distinct DN subclusters of prenatal and postnatal stage. Representative regulons are shown on the right. **(B)** Selected top enriched Gene Ontology (GO) term for DEGs of indicated cell clusters. GO term enrichment analysis was performed with Metascape. **(C)** Bar plot presenting the proportion of DN subclusters with distinct cell cycle states. Pre, Prenatal; Post, Postnatal. **(D)** Dot plot exhibiting enrichment of metabolic pathway-related GO term in indicated cell clusters. Pre, Prenatal; Post, Postnatal.

To illustrate the transcriptional differences between distinct clusters of both stages, we first identified differentially activated transcription factors (TFs) and regulons (transcriptionally coregulated operons) in each cluster by single-cell regulatory network inference and clustering (SCENIC) (31). The majority of regulon activities between clusters were varied and showed stage specificity. Prenatal clusters shared some regulons with corresponding postnatal clusters; for instance, the Non_T cluster of both prenatal and postnatal thymocytes showed higher regulon activities related to myeloid cells (CEBPD, CEBPE). Stem/progenitor cell-related regulons (HOXA10, HOXB5) were highly enriched in the ETP cluster of both stages. The activities of cell proliferation- and differentiation-related regulons (ATF3, TAF7) and T cell-related regulons (SOX5, FOXP3) were specified higher in both prenatal and postnatal T_C3 and T_C4 clusters (**Figure 3A**). Gene Ontology (GO) term analysis showed that transcripts for ETP and T_C1 cluster were enriched for terms related to the adaptive immune system, cytokine signaling in the immune system, regulation of immune effector processes, and the innate immune response, and these enrichments were more profound in postnatal clusters compared with those in the prenatal stage. The prenatal T_C2 cluster exhibited higher enrichment of GO terms related to metabolism of RNA and mRNA processing compared with corresponding postnatal clusters (**Figure 3B**). We also observed that proliferation-related GO terms (cell cycle, DNA metabolic process, and DNA replication) were enriched in prenatal DN cells as early as T_C1 cluster, but only enriched in the postnatal T_C3 and T_C4 clusters, which were congruent with the results that prenatal T_C1 and T_C2 clusters had more proliferating cells, while only postnatal T_C3 and T_C4 exhibited more active cell cycle state (**Figure 3C**). These results indicated that prenatal clusters may harbor proliferation characteristics more in advance to adapt to the rapid development of the embryo (32). Moreover, GO terms related to CD8 TCR pathway and alpha-beta T-cell activation were enriched in both postnatal T_C3 and T_C4 clusters, but only in prenatal T_C4 cluster (**Figure 3B**). These observed differences may collectively contribute to the distinct development of T cells between prenatal and postnatal stages.

Previous studies showed that thymocyte subsets exhibit distinct metabolic patterns tailored to meet the bioenergetic demands required at each developmental stage (33–36). To explore whether there were differences in metabolism between prenatal and postnatal cells, we analyzed the activities of different metabolic pathways in corresponding clusters. We found that prenatal clusters displayed much active metabolic programs related to oxidative phosphorylation, glycolysis/gluconeogenesis, pyruvate metabolism, and citrate cycle compared with the corresponding postnatal clusters, especially in ETP, T_C1, and T_C2 clusters. Arginine biosynthesis, glycerolipid metabolism, and lysine degradation, which function in activated T cells to fuel proliferation, survival, and function (37, 38), were more enriched in postnatal clusters but not prenatal cells (**Figure 3D**). These results further suggested that prenatal cells may harbor distinct metabolic characteristics to meet the bioenergetic demands required for T-cell specification.

### Differences in dynamic molecular programs between prenatal and postnatal DN cell development

**Figure 4:**
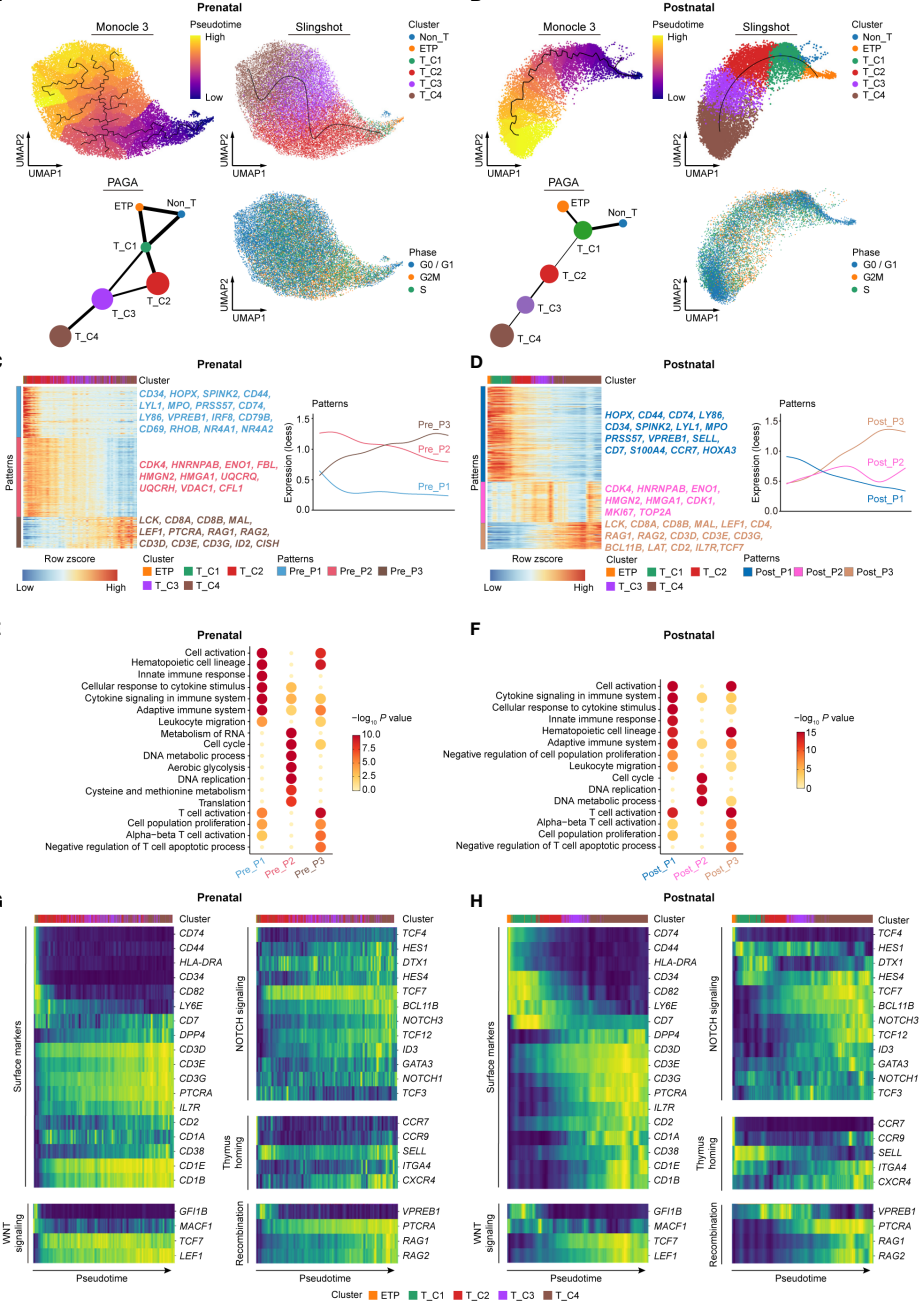
Comparing dynamic molecular programs between prenatal and postnatal DN cell development. **(A)** Developmental trajectory of prenatal DN thymocytes inferred by Monocle 3 (top left), slingshot algorithm (top right), and partition-based graph abstraction (PAGA) analysis (bottom left). UMAP for cell cycle state was shown in bottom right. **(B)** Developmental trajectory of postnatal DN thymocytes inferred by Monocle 3 (top left), slingshot algorithm (top right), and PAGA analysis (bottom left). UMAP for cell cycle state was shown in bottom right. **(C, D)** Heatmap showing the expression changes of pattern genes (top 500) along the development paths in prenatal **(C)** and postnatal **(D)** DN thymocytes. Corresponding diagrams of pattern changes were shown on the right. Pre, Prenatal; Post, Postnatal. **(E, F)** Enriched GO terms for prenatal **(E)** and postnatal **(F)** patterns. Pre, Prenatal; Post, Postnatal. **(G, H)** Heatmap exhibiting gene expression of surface markers, signaling pathways, thymus homing markers, and recombination genes involved in the T-cell differentiation ordered according to the pseudotime of the developmental trajectory for prenatal **(G)** and postnatal **(H)** DN subclusters.

We next further explored the dynamic expressional changes between prenatal and postnatal DN cells based on the developmental path. Considering the interference of batch effects in postnatal thymocyte data on the construction of the pseudo-developmental path, we therefore only chose the data with the information of immunophenotypes from Cordes et al. (12) for further analysis. Cell cycle was first corrected in all prenatal and selected postnatal DN cells to minimize the effect of proliferation on the downstream analysis. Trajectory analysis by Monocle 3 at single-cell resolution (39) revealed the developmental order of both prenatal and postnatal DN cells. Moreover, the trajectories of DN cells from both stages were also verified by slingshot algorithm (40) and partition-based graph abstraction (PAGA) analysis (**Figures 4A, B**).

Both prenatal and postnatal cells showed mainly three gene expression patterns along the developmental trajectory. Among them, the kinetics of patterns 1 and 3 were much similar between these two stages (**Figures 4C, D**). Prenatal pattern 1 enriched the genes related to hematopoietic cell lineage (*CD34*, *HOPX*, *SPINK2*, *IRF8*, and *CD79B*) and TCR signaling (*RHOB*, *NR4A1*, and *NR4A2*), and these genes were sharply downregulated across the subsequent T-cell development process. Prenatal pattern 2 included the genes associated with citric acid cycle and respiratory electron transport (*UQCRQ*, *UQCRH*) as well as DNA metabolic process (*HMGN2*, *HMGA1*), showing relatively high expression throughout the almost whole development path especially in cells enriching the T_C2 stage. Prenatal pattern 3 involved the genes gradually upregulated in the T_C3 and T_C4 clusters, in which T-cell lineage-related genes were enriched. Similar to prenatal patterns, postnatal pattern 1 enriched genes related to stem/progenitor cells (*HOPX*, *CD34*, *CCR7*, and *SPINK2*) as well as alternative lineages (*LYL1*, *MPO*, and *VPREB1*), which were sharply declined along the developmental process. Postnatal pattern 2 included proliferation- and DNA metabolism-related genes (*MKI67*, *TOP2A*, *HMGN2*, and *HMGA1*), reaching the highest expression in early and late stages of the T_C4 cluster. Compared with that in the prenatal stage, the activation of cell cycling was relatively lagging in postnatal cells. T-cell commitment-related genes were enriched in postnatal pattern 3 (**Figures 4C, D**). Next, we further performed gene functional enrichment analysis, and the results revealed that both prenatal and postnatal pattern 1 mainly expressed genes related to hematopoietic cell lineage, cell activation, cytokine signaling in immune system, innate immune response, and adaptive immune system. Cell cycle, DNA metabolic process, and DNA replication-related genes were enriched in both prenatal and postnatal pattern 2. Moreover, prenatal pattern 2 also enriched genes related to RNA metabolism, translation, aerobic glycolysis, and cysteine and methionine metabolism, which were consistent with the results observed in **Figures 3B, D**. Both prenatal and postnatal pattern 3 included genes related to T-cell activation and cell population proliferation (**Figures 4E, F**).

Finally, we focused on the transcriptional changes of a set of genes pivotal for T-cell development along the trajectory in both prenatal and postnatal thymocytes. Along the developmental order, the expression of T-cell precursor gene (*CD34*) and non-T cell gene (*HLA-DRA*, *CD74*) was dramatically decreased, which is concomitant with the activation of NOTCH signaling and WNT signaling. NOTCH signaling subsequently upregulated the expression of *TCF7*, *BCL11B*, and *GATA3*, making specification toward the T-cell lineage. The expression of *PTCRA* and *CD1A* marked full commitment of the cells to the T-cell lineage and the initiation of TCR rearrangement (16, 41). We observed that the sequential expression of these genes in postnatal cells was congruent with the reports, whereas the dynamic expression of some genes in prenatal DN cells markedly differed from those in postnatal cells. For instance, *CD7* together with some NOTCH and WNT signaling genes (*DTX1*, *HES1*, and *NOTCH1*) was highly expressed again at later stages of the T_C4 cluster, which is not the case in postnatal cells. Moreover, the expression of thymus homing genes (*SELL*, *CXCR4*, and *CCR9*) in prenatal thymocytes was also varied compared with those in postnatal cells (**Figures 4G, H**). These results indicated that prenatal thymocytes may adopt distinct gene expression patterns to meet the special demand for differentiation of T cells in embryo.

### Identification of polyadenylation site usage in human thymus

**Figure 5:**
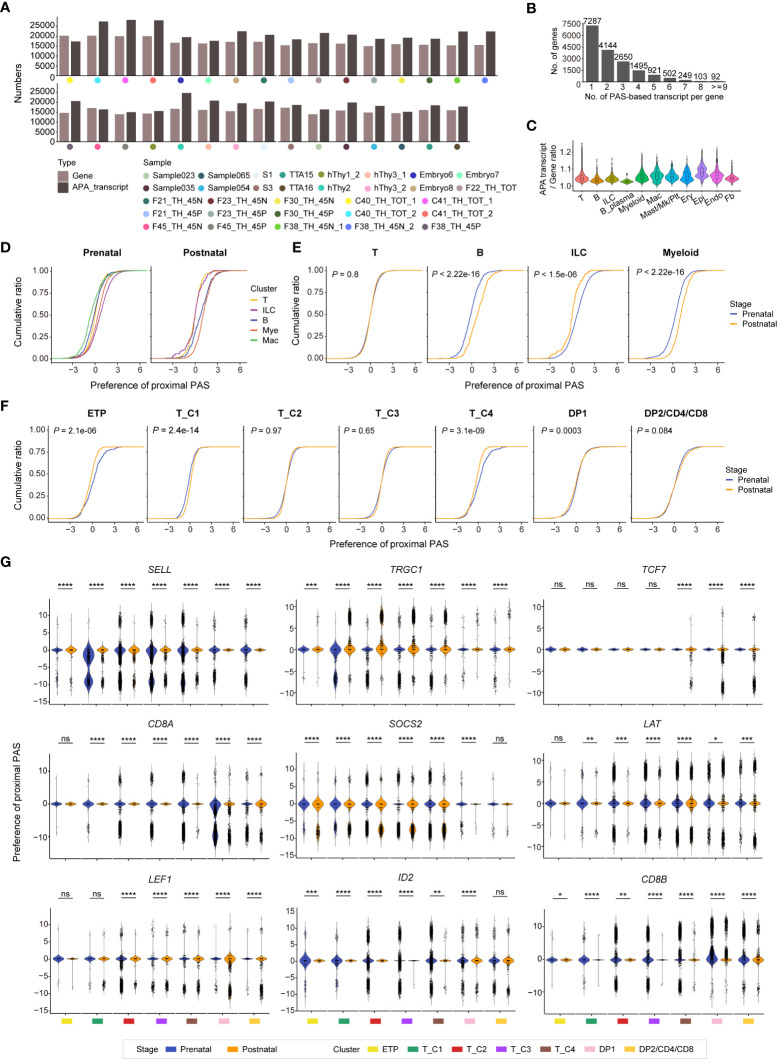
Identifying cleavage and polyadenylation sites (PASs) usages in human prenatal and postnatal thymus. **(A)** Bar plots showing the total number of genes and alternative cleavage and polyadenylation (APA) transcripts in each sample. **(B)** Bar plot showing the numbers of expressed transcripts per gene in combined 29 human thymus datasets. Each number above the bar indicates gene number. **(C)** Violin plot exhibiting the APA transcript/Gene ratio in indicated cell types of human thymus (Mac, macrophage; Mk, megakaryocyte; Plt, platelet; Ery, erythrocyte; Epi, epithelial cell; Endo, endothelial cell; Fb, fibroblast). **(D)** Characterization of proximal PAS usage in indicated cells in prenatal and postnatal thymus. **(E)** Characterization of proximal PAS usage in T, B, innate lymphoid cell (ILC), and myeloid cells in prenatal and postnatal thymus. **(F)** Characterization of proximal PAS usage in T cells (ETP, T_C1, T_C2, T_C3, T_C4, DP1, and DP2/CD4/CD8) of both prenatal and postnatal thymus. **(G)** Violin plots showing the PAS usage of indicated genes in distinct prenatal and postnatal T-cell clusters. * *P* < 0.05, ** *P* < 0.01, *** *P* < 0.001, **** *P* < 0.0001, and ns stands for not significant (Wilcoxon rank-sum test).

The majority of mammalian protein-coding genes exhibit alternative cleavage and polyadenylation (APA), resulting in mRNA isoforms with different 3’ UTRs. APA can considerably affect posttranscriptional gene regulation by alteration of the 3’ UTR length (42). The aforementioned scRNA-seq analysis has revealed the heterogeneity between human prenatal and postnatal thymus at a high resolution. The 3’ enriched strategy in library construction of most of these scRNA-seq data also allowed us to measure APA enrichment across human T-cell development. A deep learning-embedded pipeline called SCAPTURE (43) was used to capture previously unannotated cleavage and polyadenylation sites (PASs) in the human thymus. After filtering out 5’ tag-based data and quality control, 29 samples were subjected to APA analysis and the numbers of gene and APA transcript in each sample were exhibited (**Figure 5A**). Over half of the genes detected in the human thymus expressed at least two APA transcripts (**Figure 5B**), and the enrichment of APA transcripts in distinct thymic cell populations was diverse (**Figure 5C**). Thymic epithelial cells had the highest APA transcript/gene ratio, and this ratio in T cells was medium (**Figure 5C**).

For many transcripts, alternative PASs can generate multiple mRNA isoforms with different 3’ UTRs. The use of the distal PAS generates a longer 3’ UTR, while the use of proximal PAS leads to a shorter 3’ UTR. These alternative 3’ UTR isoforms allow for inclusion or exclusion of cis-regulatory elements such as RNA-binding protein sites and/or microRNA-binding sites that then result in changes in transcript abundance, stability, and translation efficiency (44). We then analyzed proximal and distal PAS usages in hematopoietic clusters of both prenatal and postnatal thymus. Compared with other prenatal cells, macrophages showed the least proximal PAS usage, whereas ILC displayed the most proximal PAS usage. While at the postnatal stage, ILC exhibited the least proximal PAS usage, and myeloid cell manifested the most proximal PAS usage (**Figure 5D**). In contrast to corresponding postnatal cells, prenatal thymic B cells and myeloid cells showed less proximal PAS usage, prenatal ILC cells exhibited more proximal PAS usage, while no preference of PAS usage was found in T cells when they were taken as a whole (**Figure 5E**). We further explored the PAS usages in detailed T-cell populations and found that the postnatal ETP and T_C4 clusters showed more preference of proximal PAS usage than corresponding prenatal clusters, while there was no/subtle difference in PAS usage in other T-cell clusters (**Figure 5F**). Next, we investigated PAS usage in several crucial T-cell lineage genes during T-cell development (**Figure 5G**). The PAS usage in each gene differed in distinct cell clusters. For instance, *TRGC1* exhibited more proximal PAS usage in postnatal thymocytes compared to prenatal cells. *SELL* and *CD8A* showed no PAS usage preference in prenatal cells but exhibited distal PAS usage in majority of the postnatal cell populations. *TCF7* and *SOCS2* manifested more distal PAS usages in postnatal cells compared with prenatal thymocytes. The distinct preference usage of PAS between prenatal and postnatal at some gene loci may affect the alternative splicing events and finally modulate gene expression level, contributing to the transcriptional heterogeneity between prenatal and postnatal thymocytes.

## Discussion

Mouse studies have well documented that there are prominent differences in sub-lineage options, differentiation speed, and proliferative yield that distinguish fetal and postnatal versions of T-cell development (15). Moreover, during embryogenesis and fetal development, the ontogeny of T cells is both multifaceted and complex, with emerging evidence suggesting that T-cell lineages arise from certain progenitor cells antedating the emergence of HSCs (45–49). However, the study of T-cell development especially during human embryogenesis has been dampened due to the scarcity of fetal material available and lack of appropriate techniques. Knowledge on early human embryonic T lymphopoiesis is vital for understanding physiology and pathological conditions and for developing immunotherapies (50). Recent advances in scRNA-seq technique have provided important new insights into the heterogeneity of T-cell precursors and the molecular mechanisms underlying T-cell lineage commitment at the single-cell level. In this study, we performed integrative transcriptomic analysis of both prenatal and postnatal thymocytes to decipher the cellular heterogeneity in human thymus, especially in CD4^–^CD8^–^DN cells. Our findings clarify that human fetal DN thymocytes exhibited distinct transcriptional and metabolic programs manifesting in elevated activities of oxidative phosphorylation and glycolysis, shedding new light on the transcriptional dynamics between prenatal and postnatal T-cell development.

Thymic T-cell differentiation occurs via a series of coordinated developmental stages that can be characterized based on cell surface marker expression. Unlike the mouse, the lack of genetic tools and the absence of defined cell surface markers have impeded our understanding of the intrathymic T-cell development in human. Although CD34, CD1a, and CD7 (51) or CD34, CD1a, and CD38 (6) were commonly used to discriminate postnatal DN cells, the identification and characterization of prenatal DN subsets are still unclear. To comprehensively understand the transcriptional characteristics of human prenatal DN cells, we fetched all prenatal and postnatal thymus cells for further analysis. Six distinct DN subpopulations were identified in both prenatal and postnatal cells. Besides T-cell fate, both prenatal and postnatal ETP/early DN cells had other lineage potential, as the ETP and T_C1 clusters also expressed alternative lineage markers (e.g., *IRF8*, *MPO*, and *SPI1*). Functional lineage potential studies of these progenitor subsets in the fetal human thymus are quite limited in the literature (7), and hence, the functional significances of cell clusters identified in the fetal human thymus via scRNA-seq remain unclear. Although we have characterized that the prenatal ETP and T_C1 clusters harbor alternative lineage potential, a key limitation of this study is that we could not obtain these two types of cells via flow cytometry sorting to validate their fate *in vitro*. Through analysis of the developmental state of these identified six populations, it was found that although prenatal and postnatal DN subclusters shared some regulons, the overall regulon activities were varied. Furthermore, prenatal and postnatal DN subclusters showed significant differences in gene expression and enriched GO terms. These discrepancies could partially explain the distinct T-cell development of two stages in human. Thymocytes with different developmental states are directed by distinct metabolic pathways and signaling networks, matching the specific functional requirements of the stage (52). These metabolic alterations in the thymus could also contribute to complex signaling mechanisms that link external signals with transcriptional events and fate decision (53, 54).

Although several groups have characterized transcriptional dynamics underlying human T-cell development (7–12), to our knowledge, it remains unclear whether exactly the same genetic mechanism functions in postnatal compared to prenatal T-cell development. We thus compared the molecular programs between prenatal and postnatal DN thymocytes along T-cell developmental paths. Prenatal and postnatal DN thymocytes exhibited three similar patterns, but the developmental characteristics of these gene patterns were very varied. Moreover, the expression dynamics of some pivotal signaling pathway genes along the T-cell development trajectory were also distinct between prenatal and postnatal thymocytes.

The majority of mammalian genes express isoforms with distinct 3’ UTRs, generated by alternative polyadenylation (APA). Since alternative 3’ UTR isoforms are involved in posttranscriptional gene regulatory mechanisms (17), it is important to explore the role of APA in development and cell fate determination. Over half of genes in the human thymus, especially in T cells, only harbored one APA transcript, which restrict us to refine single-cell clustering by using APA transcript expression. By profiling PAS usage, we indeed observed differential PAS usage in distinct cell types in both prenatal and postnatal thymocytes. Moreover, the different preference of PAS usage of several T-cell lineage genes in T-cell populations was also observed. These data provided genome-wide dynamic changes of APA during human T-cell development, providing an extra layer for comprehending T-cell development.

In summary, we have conducted an integrative transcriptomic analysis and yielded detailed information about the cellular diversity and transcriptional differences of prenatal and postnatal human thymocytes at a single-cell resolution. This study has addressed a critical question in the field regarding what are the biological differences between T-cell development in prenatal and postnatal life. Answering this question is key for understanding both T-cell biology and the strengths and limitations of lab models of human T-cell development that are based on fetal human tissue as well as their applicability to understanding postnatal T-cell development. Our study also clarifies previous unknown dynamic landscapes of APA between human prenatal and postnatal cells, providing new perspectives in the fundamental differences in prenatal versus postnatal T lymphopoiesis.

## Materials and methods

### Public datasets

Human embryonic and fetal thymus datasets were obtained from Gene Expression Omnibus (GEO; GSE133341) (7), ArrayExpress (E-MTAB-8581) (8), and National Omics Data Encyclopedia (NODE; OEP001185) (9). Human postnatal thymus datasets were obtained from GEO (GSE139042, GSE144870, and GSE195812) (10–12). The detailed information of the datasets used in this study is listed in **Supplementary Table S1**.

### Processing and quality control of sequencing data

ScRNA-seq datasets were realigned and quantified using the Cell Ranger Single-Cell Software Suite (version 6.1.3 for 3’ chemistry, 10× Genomics Inc.) with the GRCh38 human reference genome (official Cell Ranger reference, version 1.2.0). Cells with fewer than 1,000 UMI counts, 500 detected genes, and mitochondrial gene proportion >10% were considered as low-quality cells and removed from these datasets. To avoid the effects of doublets, doublets evaluated by DoubletDetection (version 4.2, https://github.com/JonathanShor/DoubletDetection) with default parameters were removed.

### Integration, dimensionality reduction, and cluster

After quality control, we used Seurat (Version 4.3.0) (55) to integrate all datasets. Genes expressed in <5 single cells were excluded. In total, we captured 187,842 cells for all prenatal and postnatal samples. Scanpy (version 1.9.1) (56) python package was used to load the cell-gene count matrix and perform downstream analysis, including data normalization (scanpy.api.pp.normalize_total method, scaling factor 10000), log-transformation (scanpy.api.pp.log1p), variable gene detection (scanpy.api.pp.highly_variable_genes), data feature scaling (scanpy.api.pp.scale), principal component analysis (PCA) (scanpy.api.tl.pca, from variable genes), batch-balanced neighborhood graph building (scanpy.external.pp.bbknn, batch_key=‘source’), and UMAP visualization (scanpy.api.ti.umap).

For clustering all cells, we used CellTypist (30) to annotate the cells with default parameters. The selected models included “Immun_All_Low” and “Immun_All_High.” As a result, we captured 29 clusters. According to the expression of feature genes, we finally summarized these clusters as ETP (HSC/MPP, ETP), T_DN, T_DP1, T_DP2/CD4/CD8, ILC (ILC, ILC precursor), B (B cells, B-cell lineage), B_plasma, Mye (DC, DC precursor, pDC, pDC precursor, granulocytes, MNP, mono-mac, monocyte precursor, monocytes, myelocytes, promyelocytes), Mac, Ery (erythrocytes, erythroid), Mast/Mk/Plt, Epi, Endo, and Fb.

Dimension reduction and subclustering were then performed within ETP and DNs in **Figure 2**. All cells from ETP and DN populations were selected for downstream analysis. After highly variable genes were calculated, we performed correlation analysis between the top 2,000 variable genes and the previously reported cell cycle genes using ‘cor’ function in R version 4.1.3. Genes with correlation coefficients greater than 0.3 and genes associated with mitochondrial, heat shock protein, and ribosome were removed. Moreover, we calculated the cell cycle gene score in each cell (scanpy.tl.score_genes_cell_cycle) and performed regression (scanpy.pp.regress_out). We also conducted regression according to the phenotype of cells to minimize the batch effect. After dimension reduction, we used Leiden graph-clustering method to conduct an unsupervised clustering. According to the expression of feature genes, we annotated clusters as Non_T, ETP, T_C1, T_C2, T_C3, and T_C4. For dimension reduction of prenatal cells in **Figure 4**, we used the same procedure. PAGA was also performed on prenatal and postnatal cells (scanpy.tl.paga).

### Differential gene expression analysis

To detect DEGs in different clusters of the datasets, we used the FindAllMarkers function in Seurat (Wilcoxon rank-sum test, with *P* value adjusted for multiple testing using Bonferroni correction). Genes with avg_logFC (log fold-change in the average expression) >log2(1.5), adjusted *P* < 0.05, and not related to mitochondria, heat shock protein, and ribosome were selected.

### Pseudotime trajectory analysis

For pseudotime trajectory analysis, we used Monocle 3 (version 1.0.0) (39) based on the previous UMAP dimension reduction from Scanpy. Pattern genes of prenatal and postnatal ETP, DN cells changing along the paths were identified by using graph_test function of Monocle 3 with its Moran’s I test, respectively. Genes with a *P* value <0.01 and the top 500 highest Moran’s I score were selected. Pattern genes were then clustered using the K-means method, and the number of clusters was determined by manually checking the heatmap results from a larger to a smaller number of clusters. We also performed pyslingshot (40) with default parameters, and the results were consistent with Monocle 3.

### Gene functional enrichment analysis

Gene functional enrichment analyses were performed using Metascape (http://metascape.org) (57). Pathways with *P* value <0.05 were selected for visualization. In addition, the R package scMetabolism (58) was used to predict the metabolism score for the cell types.

### Gene regulatory network analysis

To further evaluate the transcriptional and regulatory characteristics of T-cell subclusters (Non_T, ETP, T_C1, T_C2, T_C3, and T_C4) of different stages, gene regulatory network analysis was performed using pySCENIC (version 0.12.1) (59) following default parameters, which included gene regulatory network inference, generation of co-expression modules, regulon prediction aka cisTarget from CLI, and cellular enrichment (aka AUCell). The results were further explored in R. Only regulons with at least one other regulon and activated in at least 50% of cells in each cluster were included, and the top 10 regulons sorted by the scaled average regulon activity per cluster were chosen for visualization.

### PAS analysis

For PAS analysis, we use SCAPTURE (43) to identify, evaluate, and quantify cleavage and polyadenylation sites (PASs) from 3’ tag-based scRNA-seq. Briefly, realigned BAM files that were generated from Cell Ranger samples were used as input to call peaks for subsequent PAS evaluation. Next, DeepPASS was embedded to evaluate called peaks from 3’ tag-based scRNA-seq. Peaks with a positive prediction (predicted probability >0.5 by DeepPASS) were considered as high-confidence PASs in further analysis. Finally, UMI-tools (v1.0.1) protocol was utilized to quantify PAS-based transcripts at a single-cell level and generate barcode count matrices. The final count matrix of PAS-based transcript was analyzed in downstream single-cell tools like Seurat.

### Quantification and statistical analysis

Wilcoxon rank-sum test in R version 4.3.0 was used for DEG analysis and comparison in PAS analysis. *P* < 0.05 was considered statistically significant. The statistical details were indicated in the figure legend.

## Data availability statement

The datasets presented in this study can be found in online repositories. The names of the repository/repositories and accession number(s) can be found in the article/**Supplementary Material**.

## Ethics statement

Ethical approval was not required for the study involving humans in accordance with the local legislation and institutional requirements. Written informed consent to participate in this study was not required from the participants or the participants’ legal guardians/next of kin in accordance with the national legislation and the institutional requirements.

## Author contributions

BL and YL designed and supervised the study. HH, YY, LT, and YHL downloaded public datasets; HH performed the bioinformatics analysis with help from ZL; YY processed all figures with help from LT and YHL; YY, HH, and YL wrote the manuscript. All authors read and approved the manuscript.

## References

[1] Farley AM Morris LX Vroegindeweij E Depreter ML Vaidya H Stenhouse FH. Dynamics of thymus organogenesis and colonization in early human development. Development (2013) 140(9):2015–26. doi: 10.1242/dev.087320 PMC3631974 23571219

[2] Awong G Zúñiga-Pflücker JC. 12.09 - development of human T lymphocytes. In: McQueen CA, editor. Comprehensive toxicology, 3rd ed. Oxford: Elsevier (2018). p. 229–39.

[3] Hao QL George AA Zhu J Barsky L Zielinska E Wang X. Human intrathymic lineage commitment is marked by differential CD7 expression: identification of CD7- lympho-myeloid thymic progenitors. Blood (2008) 111(3):1318–26. doi: 10.1182/blood-2007-08-106294 PMC2214748 17959857

[4] Weerkamp F Baert MR Brugman MH Dik WA de Haas EF Visser TP. Human thymus contains multipotent progenitors with T/B lymphoid, myeloid, and erythroid lineage potential. Blood (2006) 107(8):3131–7. doi: 10.1182/blood-2005-08-3412 16384926

[5] Krangel MS. Mechanics of T cell receptor gene rearrangement. Curr Opin Immunol (2009) 21(2):133–9. doi: 10.1016/j.coi.2009.03.009 PMC2676214 19362456

[6] Dik WA Pike-Overzet K Weerkamp F de Ridder D de Haas EF Baert MR. New insights on human T cell development by quantitative T cell receptor gene rearrangement studies and gene expression profiling. J Exp Med (2005) 201(11):1715–23. doi: 10.1084/jem.20042524 PMC2213269 15928199

[7] Zeng Y Liu C Gong Y Bai Z Hou S He J. Single-cell RNA sequencing resolves spatiotemporal development of pre-thymic lymphoid progenitors and thymus organogenesis in human embryos. Immunity (2019) 51(5):930–948 e6. doi: 10.1016/j.immuni.2019.09.008 31604687

[8] Park JE Botting RA Dominguez Conde C Popescu DM Lavaert M Kunz DJ. A cell atlas of human thymic development defines T cell repertoire formation. Science (2020) 367(6480):eaay3224. doi: 10.1126/science.aay3224 32079746 PMC7611066

[9] Li Y Zeng W Li T Guo Y Zheng G He X. Integrative single-cell transcriptomic analysis of human fetal thymocyte development. Front Genet (2021) 12:679616. doi: 10.3389/fgene.2021.679616 34276782 PMC8284395

[10] Le J Park JE Ha VL Luong A Branciamore S Rodin AS. Single-cell RNA-Seq mapping of human thymopoiesis reveals lineage specification trajectories and a commitment spectrum in T cell development. Immunity (2020) 52(6):1105–1118 e9. doi: 10.1016/j.immuni.2020.05.010 32553173 PMC7388724

[11] Lavaert M Liang KL Vandamme N Park JE Roels J Kowalczyk MS. Integrated scRNA-seq identifies human postnatal thymus seeding progenitors and regulatory dynamics of differentiating immature thymocytes. Immunity (2020) 52(6):1088–1104 e6. doi: 10.1016/j.immuni.2020.03.019 32304633

[12] Cordes M Cante-Barrett K van den Akker EB Moretti FA Kielbasa SM Vloemans SA. Single-cell immune profiling reveals thymus-seeding populations, T cell commitment, and multilineage development in the human thymus. Sci Immunol (2022) 7(77):eade0182. doi: 10.1126/sciimmunol.ade0182 36367948

[13] Liu C Lan Y Liu B Zhang H Hu H. T cell development: old tales retold by single-cell RNA sequencing. Trends Immunol (2021) 42(2):165–75. doi: 10.1016/j.it.2020.12.004 33446417

[14] Zhou W Rothenberg EV. Building a human thymus: A pointillist view. Immunity (2019) 51(5):788–90. doi: 10.1016/j.immuni.2019.10.003 31747579

[15] MacNabb BW Rothenberg EV. Speed and navigation control of thymocyte development by the fetal T-cell gene regulatory network. Immunol Rev (2023) 315(1):171–96. doi: 10.1111/imr.13190 PMC10771342 36722494

[16] Rothenberg EV. Single-cell insights into the hematopoietic generation of T-lymphocyte precursors in mouse and human. Exp Hematol (2021) 95:1–12. doi: 10.1016/j.exphem.2020.12.005 33454362 PMC8018899

[17] Tian B Manley JL. Alternative polyadenylation of mRNA precursors. Nat Rev Mol Cell Biol (2017) 18(1):18–30. doi: 10.1038/nrm.2016.116 27677860 PMC5483950

[18] Di Giammartino DC Nishida K Manley JL. Mechanisms and consequences of alternative polyadenylation. Mol Cell (2011) 43(6):853–66. doi: 10.1016/j.molcel.2011.08.017 PMC3194005 21925375

[19] Derti A. A quantitative atlas of polyadenylation in five mammals. Genome Res (2012) 22(6):1173–83. doi: 10.1101/gr.132563.111 PMC3371698 22454233

[20] Tian B Manley JL. Alternative cleavage and polyadenylation: the long and short of it. Trends Biochem Sci (2013) 38(6):312–20. doi: 10.1016/j.tibs.2013.03.005 PMC3800139 23632313

[21] Elkon R Ugalde AP Agami R. Alternative cleavage and polyadenylation: extent, regulation and function. Nat Rev Genet (2013) 14(7):496–506. doi: 10.1038/nrg3482 23774734

[22] Zhang H Lee JY Tian B. Biased alternative polyadenylation in human tissues. Genome Biol (2005) 6(12):R100. doi: 10.1186/gb-2005-6-12-r100 16356263 PMC1414089

[23] Lianoglou S Garg V Yang JL Leslie CS Mayr C. Ubiquitously transcribed genes use alternative polyadenylation to achieve tissue-specific expression. Genes Dev (2013) 27(21):2380–96. doi: 10.1101/gad.229328.113 PMC3828523 24145798

[24] Singh I Lee SH Sperling AS Samur MK Tai YT Fulciniti M. Widespread intronic polyadenylation diversifies immune cell transcriptomes. Nat Commun (2018) 9(1):1716. doi: 10.1038/s41467-018-04112-z 29712909 PMC5928244

[25] Gruber AJ Zavolan M. Alternative cleavage and polyadenylation in health and disease. Nat Rev Genet (2019) 20(10):599–614. doi: 10.1038/s41576-019-0145-z 31267064

[26] Agarwal V Lopez-Darwin S Kelley DR Shendure J. The landscape of alternative polyadenylation in single cells of the developing mouse embryo. Nat Commun (2021) 12(1):5101. doi: 10.1038/s41467-021-25388-8 34429411 PMC8385098

[27] Yang Y Paul A Bach TN Huang ZJ Zhang MQ. Single-cell alternative polyadenylation analysis delineates GABAergic neuron types. BMC Biol (2021) 19(1):144. doi: 10.1186/s12915-021-01076-3 34301239 PMC8299648

[28] Cheng LC Zheng D Baljinnyam E Sun F Ogami K Yeung PL. Widespread transcript shortening through alternative polyadenylation in secretory cell differentiation. Nat Commun (2020) 11(1):3182. doi: 10.1038/s41467-020-16959-2 32576858 PMC7311474

[29] Ye C Zhou Q Hong Y Li QQ. Role of alternative polyadenylation dynamics in acute myeloid leukaemia at single-cell resolution. RNA Biol (2019) 16(6):785–97. doi: 10.1080/15476286.2019.1586139 PMC6546370 30810468

[30] Dominguez Conde C Xu C Jarvis LB Rainbow DB Wells SB Gomes T. Cross-tissue immune cell analysis reveals tissue-specific features in humans. Science (2022) 376(6594):eabl5197. doi: 10.1126/science.abl5197 35549406 PMC7612735

[31] Aibar S Gonzalez-Blas CB Moerman T Huynh-Thu VA Imrichova H Hulselmans G. SCENIC: single-cell regulatory network inference and clustering. Nat Methods (2017) 14(11):1083–6. doi: 10.1038/nmeth.4463 PMC5937676 28991892

[32] Krueger A Zietara N Lyszkiewicz M. T cell development by the numbers. Trends Immunol (2017) 38(2):128–39. doi: 10.1016/j.it.2016.10.007 27842955

[33] MacIver NJ Michalek RD Rathmell JC. Metabolic regulation of T lymphocytes. Annu Rev Immunol (2013) 31:259–83. doi: 10.1146/annurev-immunol-032712-095956 PMC3606674 23298210

[34] Almeida L Lochner M Berod L Sparwasser T. Metabolic pathways in T cell activation and lineage differentiation. Semin Immunol (2016) 28(5):514–24. doi: 10.1016/j.smim.2016.10.009 27825556

[35] Palmer CS Ostrowski M Balderson B Christian N Crowe SM. Glucose metabolism regulates T cell activation, differentiation, and functions. Front Immunol (2015) 6:1. doi: 10.3389/fimmu.2015.00001 25657648 PMC4302982

[36] Endo Y Kanno T Nakajima T. Fatty acid metabolism in T-cell function and differentiation. Int Immunol (2022) 34(11):579–87. doi: 10.1093/intimm/dxac025 35700102

[37] Kishton RJ Sukumar M Restifo NP. Arginine arms T cells to thrive and survive. Cell Metab (2016) 24(5):647–8. doi: 10.1016/j.cmet.2016.10.019 PMC6327309 27829132

[38] Gerriets VA Danzaki K Kishton RJ Eisner W Nichols AG Saucillo DC. Leptin directly promotes T-cell glycolytic metabolism to drive effector T-cell differentiation in a mouse model of autoimmunity. Eur J Immunol (2016) 46(8):1970–83. doi: 10.1002/eji.201545861 PMC5154618 27222115

[39] Cao J Spielmann M Qiu X Huang X Ibrahim DM Hill AJ. The single-cell transcriptional landscape of mammalian organogenesis. Nature (2019) 566(7745):496–502. doi: 10.1038/s41586-019-0969-x 30787437 PMC6434952

[40] Street K Risso D Fletcher RB Das D Ngai J Yosef. Slingshot: cell lineage and pseudotime inference for single-cell transcriptomics. BMC Genomics (2018) 19(1):477. doi: 10.1186/s12864-018-4772-0 29914354 PMC6007078

[41] Hosokawa H Rothenberg EV. How transcription factors drive choice of the T cell fate. Nat Rev Immunol (2021) 21(3):162–76. doi: 10.1038/s41577-020-00426-6 PMC7933071 32918063

[42] Ye C Lin J Li QQ. Discovery of alternative polyadenylation dynamics from single cell types. Comput Struct Biotechnol J (2020) 18:1012–9. doi: 10.1016/j.csbj.2020.04.009 PMC7200215 32382395

[43] Li GW Nan F Yuan GH Liu CX Liu X Chen LL. SCAPTURE: a deep learning-embedded pipeline that captures polyadenylation information from 3’ tag-based RNA-seq of single cells. Genome Biol (2021) 22(1):221. doi: 10.1186/s13059-021-02437-5 34376223 PMC8353616

[44] Arora A Goering R Lo HYG Lo J Moffatt C Taliaferro JM. The role of alternative polyadenylation in the regulation of subcellular RNA localization. Front Genet (2021) 12:818668. doi: 10.3389/fgene.2021.818668 35096024 PMC8795681

[45] Tian Y Xu J Feng S He S Zhao S Zhu L. The first wave of T lymphopoiesis in zebrafish arises from aorta endothelium independent of hematopoietic stem cells. J Exp Med (2017) 214(11):3347–60. doi: 10.1084/jem.20170488 PMC5679161 28931624

[46] Gentek R Ghigo C Hoeffel G Jorquera A Msallam R Wienert S. Epidermal gammadelta T cells originate from yolk sac hematopoiesis and clonally self-renew in the adult. J Exp Med (2018) 215(12):2994–3005. doi: 10.1084/jem.20181206 30409784 PMC6279412

[47] Boiers C Carrelha J Lutteropp M Luc S Green JC Azzoni E. Lymphomyeloid contribution of an immune-restricted progenitor emerging prior to definitive hematopoietic stem cells. Cell Stem Cell (2013) 13(5):535–48. doi: 10.1016/j.stem.2013.08.012 24054998

[48] Luis TC Luc S Mizukami T Boukarabila H Thongjuea S Woll PS. Initial seeding of the embryonic thymus by immune-restricted lympho-myeloid progenitors. Nat Immunol (2016) 17(12):1424–35. doi: 10.1038/ni.3576 PMC5172420 27695000

[49] Zhu Q Gao P Tober J Bennett L Chen C Uzun Y. Developmental trajectory of prehematopoietic stem cell formation from endothelium. Blood (2020) 136(7):845–56. doi: 10.1182/blood.2020004801 PMC7426642 32392346

[50] Sun S Wijanarko K Liani O Strumila K Ng ES Elefanty AG. Lymphoid cell development from fetal hematopoietic progenitors and human pluripotent stem cells. Immunol Rev (2023) 315(1):154–70. doi: 10.1111/imr.13197 PMC10952469 36939073

[51] Spits H. Development of alphabeta T cells in the human thymus. Nat Rev Immunol (2002) 2(10):760–72. doi: 10.1038/nri913 12360214

[52] Zhang M Lin X Yang Z Li X Zhou Z Love PE. Metabolic regulation of T cell development. Front Immunol (2022) 13:946119. doi: 10.3389/fimmu.2022.946119 35958585 PMC9357944

[53] van der Windt GJ Pearce EL. Metabolic switching and fuel choice during T-cell differentiation and memory development. Immunol Rev (2012) 249(1):27–42. doi: 10.1111/j.1600-065X.2012.01150.x 22889213 PMC3645891

[54] Gerriets VA Rathmell JC. Metabolic pathways in T cell fate and function. Trends Immunol (2012) 33(4):168–73. doi: 10.1016/j.it.2012.01.010 PMC3319512 22342741

[55] Shainer I Stemmer M. Choice of pre-processing pipeline influences clustering quality of scRNA-seq datasets. BMC Genomics (2021) 22(1):661. doi: 10.1186/s12864-021-07930-6 34521337 PMC8439043

[56] Wolf FA Angerer P Theis FJ. SCANPY: large-scale single-cell gene expression data analysis. Genome Biol (2018) 19(1):15. doi: 10.1186/s13059-017-1382-0 29409532 PMC5802054

[57] Zhou Y Zhou B Pache L Chang M Khodabakhshi AH Tanaseichuk O. Metascape provides a biologist-oriented resource for the analysis of systems-level datasets. Nat Commun (2019) 10(1):1523. doi: 10.1038/s41467-019-09234-6 30944313 PMC6447622

[58] Wu Y Yang S Ma J Chen Z Song G Rao D. Spatiotemporal immune landscape of colorectal cancer liver metastasis at single-cell level. Cancer Discovery (2022) 12(1):134–53. doi: 10.1158/2159-8290.CD-21-0316 34417225

[59] Zeisel A Munoz-Manchado AB Codeluppi S Lonnerberg P La Manno G Jureus A. Brain structure. Cell types in the mouse cortex and hippocampus revealed by single-cell RNA-seq. Science (2015) 347(6226):1138–42. doi: 10.1126/science.aaa1934 25700174

