# Supplementary material for: Divergent molecular events underlying initial T-cell commitment in human prenatal and postnatal thymus
Source: Front Immunol. 2023 Sep 27;14:1240859. doi: 10.3389/fimmu.2023.1240859 (PMC10565475; doi:10.3389/fimmu.2023.1240859)
Supplement: Supplementary file 1 [file Table_1.docx]

Supplementary Material

Divergent molecular events underlying initial T cell commitment in human prenatal and postnatal thymus

Han He^1, #^, Yingpeng Yao^1, 2, #^, Lindong Tang^1^, Yuhui Li^1^, Zongcheng Li^3^, Bing Liu^1^, ^3,^ Yu Lan^1, *^

^1^ Key Laboratory for Regenerative Medicine of Ministry of Education, Institute of Hematology, School of Medicine, Jinan University, Guangzhou, China

^2^ Basic Medicine Postdoctoral Research Station, Jinan University, Guangzhou, China

^3^ State Key Laboratory of Experimental Hematology, Haihe Laboratory of Cell Ecosystem, Institute of Hematology, Senior Department of Hematology, Fifth Medical Center of Chinese PLA General Hospital, Beijing, China

^#^ These authors contributed equally to this work.

^*^ For correspondence: Yu Lan (rainyblue_1999@126.com).

## Supplementary Tables

Supplementary Table 1. Data Information

| **Park et al., Science, 2020 (E-MTAB-8581) A cell atlas of human thymic development defines T cell repertoire formation** | | | | | | |
| --- | --- | --- | --- | --- | --- | --- |
| **Source** | **File Name** | **Sample Name**  **used in this paper** | **Age** | **Sorting strategy** | **Developmental stage** | **Used for**  **APA analysis** |
| **ERS4228662** | **WSSS8084742** | **C40_TH_TOT_1** | **PCW_7** | **total cells** | **embryo** | **Yes** |
| **ERS4228663** | **WSSS8084743** | **C40_TH_TOT_2** | **PCW_7** | **total cells** | **embryo** | **Yes** |
| **ERS4228664** | **WSSS8084744** | **C41_TH_TOT_1** | **PCW_8** | **total cells** | **embryo** | **Yes** |
| **ERS4228665** | **WSSS8084745** | **C41_TH_TOT_2** | **PCW_8** | **total cells** | **embryo** | **Yes** |
| **ERS4228629** | **FCAImmP7198432** | **F22_TH_TOT** | **PCW_9** | **total cells** | **embryo** | **Yes** |
| **ERS4228605** | **FCAImmP7198634** | **F23_TH_45P** | **PCW_11** | **CD45_P** | **embryo** | **Yes** |
| **ERS4228591** | **FCAImmP7198635** | **F23_TH_45N** | **PCW_11** | **CD45_N** | **embryo** | **Yes** |
| **ERS4228606** | **FCAImmP7579218** | **F45_TH_45P** | **PCW_12** | **CD45_P** | **embryo** | **Yes** |
| **ERS4228592** | **FCAImmP7579219** | **F45_TH_45N** | **PCW_12** | **CD45_N** | **embryo** | **Yes** |
| **ERS4228608** | **FCAImmP7528283** | **F38_TH_45P** | **PCW_13** | **CD45_P** | **embryo** | **Yes** |
| **ERS4228594** | **FCAImmP7528284** | **F38_TH_45N_1** | **PCW_13** | **CD45_N** | **embryo** | **Yes** |
| **ERS4228595** | **FCAImmP7528285** | **F38_TH_45N_2** | **PCW_13** | **CD45_N** | **embryo** | **Yes** |
| **ERS4228610** | **FCAImmP7277564** | **F30_TH_45P** | **PCW_14** | **CD45_P** | **embryo** | **Yes** |
| **ERS4228596** | **FCAImmP7277565** | **F30_TH_45N** | **PCW_14** | **CD45_N** | **embryo** | **Yes** |
| **ERS4228612** | **FCAImmP7179369** | **F21_TH_45P** | **PCW_16** | **CD45_P** | **embryo** | **Yes** |
| **ERS4228613** | **FCAImmP7179370** | **F21_TH_45N** | **PCW_16** | **CD45_N** | **embryo** | **Yes** |
| **Zeng et al., Immunity, 2019 (GSE133341) Single-Cell RNA Sequencing Resolves Spatiotemporal Development of Pre-thymic Lymphoid Progenitors and Thymus Organogenesis**  **in Human Embryos** | | | | | | |
| **Source** | **File Name** | **Sample Name**  **used in this paper** | **Age** | **Sorting strategy** | **Developmental stage** | **Used for**  **APA analysis** |
| **SRX6367763** | **GSM3906003** | **Embryo6** | **PCW_8** | **CD235a-** | **embryo** | **Yes** |
| **SRX6367765** | **GSM3906005** | **Embryo7** | **PCW_9** | **CD235a-CD45-(30%) CD235a-CD45+(70%)** | **embryo** | **Yes** |
| **SRX6367764** | **GSM3906004** | **Embryo8** | **PCW_10** | **CD235a-CD45-(30%) CD235a-CD45+(70%)** | **embryo** | **Yes** |
| **Li et al., Front Genet, 2021 (OEP001185) Integrative Single-Cell Transcriptomic Analysis of Human Fetal Thymocyte Development** | | | | | | |
| **Source** | **File Name** | **Sample Name**  **used in this paper** | **Age** | **Sorting strategy** | **Developmental stage** | **Used for**  **APA analysis** |
| **OER087537** | **OES052221** | **Sample035** | **PCW_9** | **total live cells** | **embryo** | **Yes** |
| **OER087534** | **OES052222** | **Sample054** | **PCW_11** | **total live cells** | **embryo** | **Yes** |
| **OER087536** | **OES052223** | **Sample023** | **PCW_13** | **total live cells** | **embryo** | **Yes** |
| **OER087535** | **OES052224** | **Sample065** | **PCW_15** | **total live cells** | **embryo** | **Yes** |
| **Lavaert et al., Immunity, 2020 (GSE144870) Integrated scRNA-Seq Identifies Human Postnatal Thymus Seeding Progenitors and Regulatory Dynamics of**  **Differentiating Immature Thymocytes** | | | | | | |
| **Source** | **File Name** | **Sample Name**  **used in this paper** | **Age** | **Sorting strategy** | **Developmental stage** | **Used for**  **APA analysis** |
| **SRX7691514** | **GSM4299785** | **S1** | **2-24months** | **Lin-CD34+CD1a-** | **Postnatal** | **Yes** |
| **SRX7691515** | **GSM4299786** | **S2** | **2-24months** | **Lin-CD34+CD1a-** | **Postnatal** | **No** |
| **SRX7691516** | **GSM4299787** | **S3** | **2-24months** | **Lin-CD34+CD1a-** | **Postnatal** | **Yes** |
| **SRX7691517** | **GSM4299788** | **TTA15** | **2-24months** | **CD44+** | **Postnatal** | **Yes** |
| **SRX7691518** | **GSM4299789** | **TTA16** | **2-24months** | **CD44+** | **Postnatal** | **Yes** |
| **Le et al., Immunity, 2020 (GSE139042) Single-Cell RNA-Seq Mapping of Human Thymopoiesis Reveals Lineage Specification Trajectories and a Commitment Spectrum**  **in T Cell Development** | | | | | | |
| **Source** | **File Name** | **Sample Name**  **used in this paper** | **Age** | **Sorting strategy** | **Developmental stage** | **Used for**  **APA analysis** |
| **SRX7015013** | **GSM4127993** | **hThy1_1** | **19 months** | **CD34+CD4-CD8-GlyA-** | **Postnatal** | **No** |
| **SRX7015014** | **GSM4127994** | **hThy1_2** | **19 months** | **CD34-CD45+GlyA-** | **Postnatal** | **Yes** |
| **SRX8241228** | **GSM4505165** | **hThy2** | **23 months** | **CD34+CD4-CD8-GlyA-** | **Postnatal** | **Yes** |
| **SRX8241229** | **GSM4505166** | **hThy3_1** | **9 days** | **CD34+CD4-CD8-GlyA-** | **Postnatal** | **Yes** |
| **SRX8241230** | **GSM4505167** | **hThy3_2** | **9 days** | **CD34-CD45+GlyA-** | **Postnatal** | **Yes** |
| **SRX8241234** | **GSM4505171** | **hThy7** | **5 years** | **CD34-CD45+GlyA-** | **Postnatal** | **No** |
| **Cordes et al., Sci Immunol, 2022 (GSE195812) Single-cell immune profiling reveals thymus-seeding populations, T cell commitment, and multilineage development in the human thymus** | | | | | | |
| **Source** | **File Name** | **Sample Name**  **used in this paper** | **Age** | **Sorting strategy** | **Developmental stage** | **Used for**  **APA analysis** |
| **SRX13998815** | **GSM5851294** | **DN1** | **7 weeks to 3 years** | **CD34+CD38-CD1a** | **Postnatal** | **No** |
| **SRX13998816** | **GSM5851295** | **DN2** | **7 weeks to 3 years** | **CD34+CD38+CD1a-** | **Postnatal** | **No** |
| **SRX13998817** | **GSM5851296** | **DN3** | **7 weeks to 3 years** | **CD34-CD38+CD1a+** | **Postnatal** | **No** |
| **SRX13998818** | **GSM5851297** | **ISP** | **7 weeks to 3 years** | **CD3lowCD4+** | **Postnatal** | **No** |
| **SRX13998819** | **GSM5851298** | **DP_CD3min** | **7 weeks to 3 years** | **CD4+CD8+CD3-** | **Postnatal** | **No** |
| **SRX13998820** | **GSM5851299** | **DP_CD3plus** | **7 weeks to 3 years** | **CD4+CD8+CD3+** | **Postnatal** | **No** |
| **SRX13998821** | **GSM5851300** | **CD4** | **7 weeks to 3 years** | **CD3-CD4+CD8-** | **Postnatal** | **No** |
| **SRX13998822** | **GSM5851301** | **CD8** | **7 weeks to 3 years** | **CD3+CD4-CD8+** | **Postnatal** | **No** |

Supplementary Table 2. Integrated annotation of each cell for human thymus from different published datasets

Supplementary Table 3. Subcluster annotation of each cell from ETP and T_DN clusters in Fig. 1

Supplementary Table 4. Scaled regulon activity for prenatal and postnatal clusters

Supplementary Table 5. UMAP dimensionality reduction for prenatal cells

Supplementary Table 6. Pseudotime inferred by Monocle 3 for prenatal and postnatal cells

Supplementary Table 7. Genes for prenatal and postnatal patterns

**Supplementary Table 2 to Supplementary Table 7 can be accessed at https://doi.org/10.6084/m9.figshare.24056592.**
